# A Robust Extended Kalman Filter Algorithm Based on a Sliding Window Fractional-Order Grey Prediction Model and Its Application in MINS/GNSS

**DOI:** 10.3390/s26061836

**Published:** 2026-03-14

**Authors:** Mingze Zhang, Aigong Xu

**Affiliations:** School of Geomatics, Liaoning Technical University, Fuxin 123000, China; xuaigong@lntu.edu.cn

**Keywords:** MINS/GNSS, robust extended Kalman filter, sliding window, fractional-order grey prediction model, weighted index SPRT

## Abstract

To address the issue of reduced accuracy or even divergence in micro-electro-mechanical inertial navigation systems’/global navigation satellite systems’ (MINSs’/GNSSs’) integrated navigation systems caused by small amplitude fault in GNSS measurement information, this paper proposes a robust extended Kalman filter algorithm based on a sliding window fractional-order grey prediction model (SWFGM(1,1)-REKF). When GNSS signals are disrupted, this algorithm first detects system faults through a weighted index sequential probability ratio test (SPRT) detection. Then, it uses GNSS measurements predicted by a sliding window fractional-order grey prediction model (FGM(1,1)) to replace the faulty GNSS data and integrates them with MINSs. Finally, it combines robust estimation to construct a robust extended Kalman filter to correct the integrated information. Simulation and vehicle experiment results show the advancement of SWFGM(1,1)-REKF. When GNSS measurements experience small amplitude abrupt faults, compared with traditional robust extended Kalman filter algorithm based on a chi-square test, the proposed algorithm improves filtering accuracy of velocity and position. In the vehicle small amplitude mutation fault experiment, the velocity and position accuracy are increased by more than 50% and 80% respectively.

## 1. Introduction

The inertial measurement unit (IMU) based on a micro-electro-mechanical system (MEMS) has developed rapidly due to its small size and low power consumption. Micro-electro-mechanical inertial navigation systems/global navigation satellite systems (MINSs/GNSSs) have been widely applied in many fields such as mobile measurement, intelligent transportation, and vehicle navigation [1,2]. However, when GNSS is used in complex environments such as cities, canyons, and areas with electromagnetic radiation, the signal is disturbed and prone to failure [3,4]. This causes the accuracy of MINS/GNSS to decrease rapidly. Therefore, processing GNSS measurement fault information and enhancing the fault tolerance of the system are particularly important for improving the accuracy of MINS/GNSS [5,6].

The key to improving the fault tolerance of the system is to detect the system fault quickly and deal with it effectively. Currently, there are many methods for processing after GNSS fault detection, but there are few methods for processing during GNSS faults. The commonly fault-tolerant methods include INS independent solution by isolating GNSS fault observations, a filtering algorithm based on interacting multiple models and a robust adaptive filtering method based on a hypothesis test [7,8,9]. However, for low-precision MINS/GNSS, the long-term independent solution of INS will cause significant error accumulation, resulting in a rapid decline or even divergence of navigation accuracy. In the filtering algorithm based on interacting multiple models, since the system model is not completely known, the constructed model does not necessarily contain the current system model, which limits the accuracy improvement of MINS/GNSS [10,11]. A robust adaptive filtering method based on a hypothesis test reduces the weight of fault observations by calculating the equivalent weight matrix, thereby reducing the impact of faults on system accuracy [12,13,14]. Reference [15] proposes a robust extended Kalman filter (REKF) based on the sequential chi-square test. It calculates equivalent weight factors through a fault detection function and adjusts the noise covariance matrix in real time to improve navigation accuracy. At the same time, EKF filtering can also avoid the interference caused by nonlinear errors. Compared with ordinary EKF, the state estimation accuracy is more accurate [16,17,18]. However, the accuracy of such methods depends on the detection statistics. When the detection statistic’s value is large, the robust effect is good. When the detection statistic’s value is small, the robust effect is poor.

In recent years, methods based on time series prediction, such as machine learning and grey prediction models, have been widely used in integrated navigation systems [19,20]. Among them, the time series prediction method based on machine learning requires a large amount of sample data for model training [21,22,23]. If the number of training samples is small, the prediction accuracy will be poor, and the ideal effect will not be achieved. Among them, the grey prediction model [24] has a small amount of data and high prediction accuracy in a short time. Therefore, it has received extensive attention. Currently, the commonly used grey prediction model is the GM(1,1) model. The GM(1,1) model simplifies the randomness of the data and excavates the potential rules behind the data by processing the original data. Then, a differential equation model is established to predict. Finally, the prediction results are obtained by inverse operation. But the model has insufficient ability to deal with nonlinear sequences. In order to enhance the processing ability of nonlinear sequences, many scholars have introduced the idea of fractional-order into the processing of integrated navigation data. Reference [25] introduced the fractional-order idea on the basis of the grey model to construct the fractional-order grey prediction model. This model extends the first-order accumulation generation of the traditional grey model to the fractional-order accumulation generation, and finds the most suitable accumulation order by optimization, so that the processed data can present the exponential law more perfectly, so as to construct a more accurate differential equation model for prediction. This model has higher-order flexibility, can describe the inherent characteristics of data more flexibly and more accurately, and can better deal with nonlinear sequence data. However, the model does not update the data. With the increase in fault data, more and more fault data are involved in the calculation, which leads to a decrease in model prediction accuracy. So, in order to reduce the influence of fault data on the MINS/GNSS, enhance the fault tolerance of the system and improve the estimation accuracy of the system, we propose a robust extended Kalman filter algorithm based on a sliding window fractional-order grey prediction model (SWFGM(1,1)-REKF). The algorithm combines fault detection, data prediction and robust filtering to form a closed-loop combined fault data processing flow. First, the algorithm constructs a weighted index sequential probability ratio test algorithm based on the sequential probability ratio test (SPRT) to detect system faults. Secondly, the FGM(1,1) model is introduced, and the sliding window FGM(1,1) model is constructed to predict the GNSS observation information at the current time. The GNSS fault data is replaced and then combined with MINS, which greatly eliminates the fault data and reduces the influence of the fault data on the EKF filter. Finally, the algorithm calculates the equivalent weight factor according to the fault detection function value, constructs the equivalent weight covariance matrix of the observation vector, and applies the equivalent weight covariance matrix to the extended Kalman filter to further reduce the influence of the fault data, thereby improving the accuracy and robustness of the system state estimation.

### 1.1. MINS/GNSS Model

In the MINS/GNSS, the parameter error output by the navigation subsystem is used as the state variable of the integrated navigation system. The difference between the navigation parameters calculated by MINS and the navigation information output by the GNSS receiver is used as the measurement input of MINS/GNSS. The error model of MINS is used as the system equation of MINS/GNSS [26,27]. In this paper, the east-north-up coordinate system is selected as the navigation coordinate system. The loose combination was selected as the system combination. In the MINS/GNSS loosely integrated navigation model, the system state equation is obtained by selecting 15-dimensional state parameters. The state variable can be expressed as:

(1) $$X={\left[\phi ,\delta v^{n},\delta p^{n},\varpi^{b},\nabla^{b}\right]}^{T}$$

where ϕ is the attitude error of the carrier, $\phi =[\begin{array}{ccc} \phi_{E} & \phi_{N} & \phi_{U} \end{array}]$, $\delta v^{n}$ is the velocity error of the carrier, $\delta v^{n}=[\delta v_{E}\;\delta v_{N}\;\delta v_{U}]$, $\delta p^{n}$ is the position error of the carrier, $\delta p^{n}=\begin{array}{ccc} {}[\delta L & \delta \lambda & \delta h \end{array}]$, $\varepsilon^{b}$ is the bias of the gyroscope, $\varpi^{b}=[\varpi_{x}\;\varpi_{y}\;\varpi_{z}]$, and $\nabla^{b}$ is the bias of the accelerometer, $\nabla^{b}=[\nabla_{x}\;\nabla_{y}\;\nabla_{z}]$.

The system state equation of MINS/GNSS after discretization can be expressed as:

(2) $$X_{k}=f(X_{k-1})+\Gamma_{k-1}W_{k-1}$$

where $X_{k}$ is the 15-dimensional system state vector at time k, $f\left(\cdot \right)$ is 15-dimensional nonlinear state transition function. In the calculation of the system equation, the first-order Taylor expansion is performed around the filter value, $\Gamma_{k-1}$ is the 15 × 6 system noise matrix, $W_{k-1}$ is the 6-dimensional system noise matrix at previous time k−1, the noise conforms to the normal distribution, and the corresponding noise matrix is $Q_{k}$.

The measurement equation of the MINS/GNSS after discretization can be expressed as:

(3) $$Z_{k}=h(X_{k})+V_{k}$$

where $Z_{k}$ is the measurement matrix at time k, $h\left(\cdot \right)$ is a nonlinear observation matrix, in the calculation of the system equation, the first-order Taylor expansion is performed around the filter value, and $V_{k}$ is the measurement noise matrix at time k.

The discrete EKF can be expressed as:

(4) $$\left\{\begin{array}{l} {\hat{X}}_{k/k-1}=f({\hat{X}}_{k-1})\\ P_{k/k-1}=\Phi_{k/k-1}P_{k-1}\Phi_{k.k-1}^{T}+\Gamma_{k/k-1}Q_{k-1}\Gamma_{k/k-1}^{T}\\ K_{k}=P_{k/k-1}H_{k}^{T}{\left(H_{k}P_{k/k-1}H_{k}^{T}+R_{k}\right)}^{-1}\\ P_{k}=\left(I-K_{k}H_{k}\right)P_{k/k-1}\\ {\hat{X}}_{k}={\hat{X}}_{k/k-1}+K_{k}\left(Z_{k}-h({\hat{X}}_{k/k-1})\right) \end{array}\right.$$

where $\Phi_{k/k-1}=J(f({\hat{X}}_{k-1}))$ is the state transition matrix, $H_{k}=J(h({\hat{X}}_{k-1}))$ is the observation matrix, J(⋅) is the Jacobian matrix, ${\hat{X}}_{k/k-1}$ is the state one-step prediction from time k−1 to time k, $P_{k/k-1}$ is the state one-step prediction covariance matrix from time k−1 to time k, $K_{k}$ is the filter gain matrix at time k, ${\hat{X}}_{k}$ is the state estimation at time k, $P_{k}$ is the state estimation covariance matrix at time k, $Q_{k}$ is the system noise covariance matrix at time k, and $R_{k}$ is the measurement noise covariance matrix at time k.

### 1.2. Weighted Index SPRT Method

In integrated navigation system, the chi-square test method is commonly used to detect system faults. However, this method has the problems of false detection and missed detection for small amplitude fault detection. In order to accurately and quickly detect small amplitude faults, this paper proposes a weighted index SPRT method.

In EKF, the innovation can be expressed as:

(5) $$\varepsilon_{k}=Z_{k}-h({\hat{X}}_{k/k-1})$$

Then the i-dimensional filtering innovation vector $\varepsilon_{i,k}$ at time k can be expressed as:

(6) $$\varepsilon_{i,k}=Z_{i,k}-h_{i}({\hat{X}}_{i,k/k-1})$$

where $Z_{i,k}$ is the i measurement vector at time k, ${\hat{X}}_{i,k/k-1}$ is the one-step prediction state of the i-dimensional from time k−1 to time k.

Since the difference between the MINS/GNSS is used as the measurement input of the MINS/GNSS. i is an integer, and 1≤i≤6.

The i-dimensional filtering innovation vector at moments 1~k are selected to form the innovation sequence. From the probability theory and mathematical statistics, it can be obtained that $\varepsilon ∼N({\bar{\varepsilon }}_{i,k},D_{i,k})$. ${\bar{\varepsilon }}_{i,k}$ is the expected value of the i-dimensional innovation. $D_{i,k}$ represents the expected value of the i-dimensional variance [13]. It can be seen from the above:

(7) $$Ε[\varepsilon_{i,k}]={\bar{\varepsilon }}_{i,k},\;Ε[\varepsilon_{i,k}\varepsilon_{i,k}^{T}]=D_{i,k}$$

(8) $$D_{i,k}=H_{i,k}P_{i,k/k-1}H_{i,k}^{T}+R_{i,k}$$

where $H_{i,k}$ is the observation matrix of the i-dimension at time k. $R_{i,k}$ is the i-dimensional measurement noise covariance matrix at time k.

When there is no fault in GNSS measurement data, ${\bar{\varepsilon }}_{i,k}=0$. When there is fault in GNSS measurement data, ${\bar{\varepsilon }}_{i,k}\neq 0$ [13]. Hypothesis testing: $H_{0}:{\bar{\varepsilon }}_{i,k}=0$, it is that the system has no fault, $H_{1}:{\bar{\varepsilon }}_{i,k}\neq 0$, it is that the system has fault.

The likelihood function $p\left(\cdot \right)$ of the innovation vector constructed according to the hypothesis can be expressed as:

(9) $$p\left(\varepsilon_{i,k}|H_{0}\right)=\frac{1}{2\pi^{n/2}{\left(D_{i,k}\right)}^{1/2}}e^{-\frac{1}{2}\varepsilon_{i,k}^{T}{\left(D_{i,k}\right)}^{-1}\varepsilon_{i,k}}$$

(10) $$p\left(\varepsilon_{i,k}|H_{1}\right)=\frac{1}{2\pi^{n/2}{\left(D_{i,k}\right)}^{1/2}}e^{-\frac{1}{2}{\left(\varepsilon_{i,k}-{\bar{\varepsilon }}_{i,k}\right)}^{T}{\left(D_{i,k}\right)}^{-1}\left(\varepsilon_{i,k}-{\bar{\varepsilon }}_{i,k}\right)}$$

According to the likelihood function, the likelihood ratio $L_{k}$ can be expressed as:

(11) $$L_{k}=\prod_{j=1}^{k}\frac{p\left(\varepsilon_{i,j}|H_{1}\right)}{p\left(\varepsilon_{i,j}|H_{0}\right)}=e^{\overset{k}{\underset{j=1}{\Sigma }}\frac{\varepsilon_{i,j}^{2}-{\left(\varepsilon_{i,j}-{\bar{\varepsilon }}_{i,j}\right)}^{2}}{2D_{i,j}}}$$

By taking logarithms on both sides, the detection function of SPRT can be expressed as:

(12) $$\lambda_{k}=\lambda_{k-1}+\frac{2\varepsilon_{i,k}{\bar{\varepsilon }}_{i,k}^{T}-{\bar{\varepsilon }}_{i,k}{\bar{\varepsilon }}_{i,k}^{T}}{2}D_{i,k}^{-1}$$

where $\lambda_{k}$ represents the fault detection function. According to Formula (13)

(13) $${\bar{\varepsilon }}_{i,k}=\frac{1}{k}\sum_{j=1}^{k}\varepsilon_{i,j}=\frac{\left(k-1\right){\bar{\varepsilon }}_{i,k-1}+\varepsilon_{i,k}}{k}$$

The statistical change rate of the SPRT can be expressed as:

(14) $$\Delta \lambda_{k}=\frac{2\varepsilon_{i,k}{\bar{\varepsilon }}_{i,k}^{T}-{\bar{\varepsilon }}_{i,k}{\bar{\varepsilon }}_{i,k}^{T}}{2}D_{i,k}^{-1}$$

According to the modified Equations (12)–(14), we can see that in order to detect the end time of the fault, the statistic $\lambda_{k}$ needs to fall below the detection threshold quickly at the end of the fault. However, after the end of the fault, when the measurement system returns to normal, ${\bar{\varepsilon }}_{i,k}$ tends to zero, which leads to the change rate of statistics $\Delta \lambda_{k}$ also tends to zero. Finally, after the end of the fault, the statistic $\lambda_{k}$ is always greater than the threshold, and the end of the fault cannot be detected. Therefore, in order to accurately detect the end time of the fault, we use the exponential weighted method to calculate the expected value of the innovation. Let

(15) $$\left\{\begin{array}{c} \sum_{j=1}^{k}\beta_{j}=1\\ \beta_{j-1}=\beta_{j}c \end{array}\right.\;0<c<1$$

where $\beta_{i}$ represents the weighted coefficient. c is the forgetting factor. The earlier the data, the smaller the weighting coefficient.

The weighted coefficient constructed by can be expressed as:

(16) $$\beta_{j}=\frac{1-c}{1-c^{k}}c^{k-i}\;j=1,2\cdots k$$

Then the expected value ${\bar{\varepsilon }}_{i,k}$ of the innovation vector constructed by the exponential weighted recursive estimation method can be expressed as:

(17) $${\bar{\varepsilon }}_{i,k}=\sum_{j=1}^{k}\beta_{j}\left(H_{i,k}{\tilde{X}}_{i,k/k-1}+V_{i,k}\right)=\left(1-d_{k}\right){\bar{\varepsilon }}_{i,k-1}+d_{k}\varepsilon_{i,k}$$

where ${\tilde{X}}_{i,k/k-1}=X_{i,k}-{\hat{X}}_{i,k/k-1}$ is the one-step prediction state vector error of the i-dimension at time k. $V_{i,k}$ denotes the i-dimension measurement noise at time k. In the calculation of the estimated innovation in the expected value of innovation, c is 0.75.

Similarly, the estimated innovation vector covariance matrix can be expressed as:

(18) $${\hat{D}}_{i,k}=d_{k}({\tilde{Z}}_{i,k/k-1}{\tilde{Z}}_{i,k/k-1}^{T})+(1-d_{k}){\hat{D}}_{i,k-1}$$

where ${\tilde{Z}}_{i,k/k-1}=Z_{i,k}-{\hat{Z}}_{i,k/k-1}$ is the one-step prediction measurement vector error of the i-dimension at time k.

In order to eliminate the deviation between ${\hat{D}}_{i,k}$ and $D_{i,k}$ in fault innovation mismatch. We define a deviation adjustment function $\alpha_{i,k}$. According to $\alpha_{i,k}$, we can get,

(19) $${\hat{D}}_{i,k}=\alpha_{i,k}D_{i,k}$$

Then the deviation adjustment function is calculated as follows:

(20) $$\alpha_{i,k}=\min\left\{1,tr({\hat{D}}_{ik})/tr(D_{i,k})\right\}$$

By introducing the calculated deviation adjustment function into the detection function, the fault detection statistic function after exponential weighting can be expressed as:

(21) $$\lambda_{k}=\alpha_{i,k}\lambda_{k-1}+\frac{2\varepsilon_{k}{\bar{\varepsilon }}_{k}-{\bar{\varepsilon }}_{k}^{2}}{2D_{i,k}}$$

Set the detection threshold $T_{\mathrm{down}}$

(22) $$T_{\mathrm{down}}=\ln(\left(1-P_{m}\right)/P_{f})$$

where $P_{m}$ is the missed alarm rate and $P_{f}$ is the false alarm rate. When $\lambda_{k}>T_{\mathrm{down}}$, it is determined that the system has an unknown fault. When $\lambda_{k}\leq T_{\mathrm{down}}$, it is determined that the system is no fault. In this paper, $P_{m}$ is set to 0.035, $P_{f}$ is $8\times 10^{-6}$ [28], and the calculated threshold is 11.7.

## 2. SWFGM(1,1)-REKF Algorithm

### 2.1. Construction of SWFGM(1,1) Model

This method is designed to reduce the interference of fault data to the MINS/GNSS system and improve the accuracy of the system. Combined with the literature [24], we construct the SWFGM(1,1) model to predict the GNSS measurement information at the fault time. Then, the predicted GNSS velocity, position, and other measurement information are used to replace the GNSS velocity and position information with faults to improve the accuracy of MINS/GNSS. The SWFGM(1,1) model is constructed as follows:

It is assumed that the historical data sequence of GNSS measurements used for prediction can be expressed as:

(23) $$x^{\left(0\right)}=\left(x^{\left(0\right)}(1),x^{\left(0\right)}(2),\cdots ,x^{\left(0\right)}(n)\right)$$

where x is the sequence, x is a certain data, $x^{\left(0\right)}$ is the data is not calculated by addition, and is a collection of original data.

After the Formula (23) is cumulatively added r times, the expression of an item in the determinant can be expressed as:

(24) $$x^{\left(r\right)}(k)=\sum_{i-1}^{k}C_{k-i+r-1}^{k-i}x^{\left(0\right)}(i)$$

(25) $$C_{k-i+r-1}^{k-i}=\frac{(k-i+r-1)(k-i+r-2)\cdots (r+1)r}{(k-i)!}$$

The *r*-order accumulation sequence can be expressed as:

(26) $$x^{\left(r\right)}=\left(x^{\left(r\right)}(1),x^{\left(r\right)}(2),\cdots ,x^{\left(r\right)}(n)\right)$$

According to the r-order cumulative sequence, the adjacent mean sequence $z^{\left(r\right)}$ can be expressed as [29]:

(27) $$z^{\left(r\right)}(k)=\frac{1}{2}\left(x^{\left(r\right)}(k)+x^{\left(r\right)}(k-1)\right)$$

where $z^{\left(r\right)}(k)$ is the adjacent mean value at time k after r times addition.

The FGM(1,1) grey differential equation and its whitening differential equation can be expressed as:

(28) $$x^{\left(0\right)}(k)+az^{\left(r\right)}(k)=b$$

(29) $$\frac{dx^{\left(r\right)}(k)}{dk}+ax^{\left(r\right)}(k)=b$$

where a is the development coefficient and b is the grey action quantity. Equation (28) is written as a matrix.

(30) $$Y=B\hat{\alpha }$$

where

(31) $$Y=\left[\begin{array}{c} x^{\left(r\right)}(2)-x^{\left(r\right)}(1)\\ x^{\left(r\right)}(3)-x^{\left(r\right)}(2)\\ \vdots \\ x^{\left(r\right)}(n)-x^{\left(r\right)}(n-1) \end{array}\right]$$

(32) $$B=\left[\begin{array}{cc} -z^{\left(r\right)}(2) & 1\\ -z^{\left(r\right)}(3) & 1\\ \vdots & \vdots \\ -z^{\left(r\right)}(n) & 1 \end{array}\right]$$

(33) $$\hat{\alpha }=\left[\begin{array}{c} a\\ b \end{array}\right]$$

According to the least squares method, we can get

(34) $$\hat{\alpha }={\left(B^{T}B\right)}^{-1}B^{T}Y$$

Substitute $\hat{\alpha }$ into Equation (28). Let $x^{\left(r\right)}(0)=x^{\left(0\right)}(1)$. The time response function can be expressed as [29]:

(35) $${\hat{x}}^{\left(r\right)}(t)=(x^{\left(0\right)}(t_{0})-b/a)e^{-a(t-t_{0})}+b/a$$

where $t_{0}$ is the initial time.

Therefore, the time response sequence of the FGM(1,1) grey differential equation can be expressed as:

(36) $${\hat{x}}^{\left(r\right)}(k+1)=(x^{\left(0\right)}(1)-b/a)e^{-ak}+b/a$$

where $k=\begin{array}{cccc} 1, & 2, & \cdots , & n \end{array}$. According to Equation (30), the fitting value sequence can be expressed as:

(37) $${\hat{x}}^{\left(r\right)}=\left({\hat{x}}^{\left(r\right)}(1),{\hat{x}}^{\left(r\right)}(2),\cdots ,{\hat{x}}^{\left(r\right)}(n),\cdots \right)$$

In Equation (36), the data before time k+1 is a priori sequence, and the data after time k+1 is a prediction sequence. Based on the prediction model, the predicted values are obtained by inverse generation:

(38) $${\hat{x}}^{\left(0\right)}(k)=\beta^{\left(r\right)}{\hat{x}}^{\left(r\right)}={\hat{x}}^{\left(r)(1-r\right)}(k)-{\hat{x}}^{\left(r)(1-r\right)}(k-1)$$

where ${\hat{x}}^{\left(0\right)}(k+1)$ can be calculated using Equation (30). Add ${\hat{x}}^{\left(0\right)}(k+1)$, delete $x^{\left(0\right)}(1)$, and construct a new sequence:

(39) $$\left\{x^{\left(0\right)}(2),x^{\left(0\right)}(3),\cdots ,{\hat{x}}^{\left(0\right)}(k+1)\right\}$$

Perform the operation of above Equations (37)–(39), we can get ${\hat{x}}^{\left(0\right)}(k+2)$. And so on, we get ${\hat{x}}^{\left(0\right)}(k+n)$.

In the FGM(1.1) model, the cumulative addition times of the data sequence have a crucial influence on the accuracy of the prediction results. In order to find the optimal cumulative addition number of the sequence, this paper uses the particle swarm optimization algorithm to find the optimal number. The particle swarm optimization algorithm is relatively simple and has fast convergence speed. It is an intelligent optimization algorithm inspired by the foraging behavior of birds. In the particle swarm optimization algorithm, the fitness function determines the accuracy of the solution, so its selection is very important [30]. In this paper, the average relative minimum error is used as the fitness function, and the optimal cumulative number of additions is determined by calculating the fitness value. The number of particles is set to 20, and the maximum number of iterations is 300. When the average relative error is minimized, the algorithm optimization stops and the iteration ends. The fitness function can be expressed as:

(40) $$\min f=\frac{1}{n}\sum_{i=1}^{n}\left|\frac{{\hat{x}}^{\left(0\right)}(i)-x^{\left(0\right)}(t-8+i,j)}{x^{\left(0\right)}(t-8+i,j)}\right|100$$

where n represents the number of original data sequences. After multiple experiments, the original data is 7, and the size of the sliding window is set to 7, t represents the fault initiation time, j represents the fault dimension, and ${\hat{x}}^{\left(0\right)}(i)$ represents the original data prediction values of the GNSS fault dimension, $x^{\left(0\right)}$ represents the original data of the GNSS fault dimension.

### 2.2. SWFGM(1,1)-REKF Flow Chart

When the system has a fault, the GNSS observations predicted by the sliding window FGM(1,1) model are affected by fault information. In order to reduce the influence of measurement fault information on state estimation, the proportion of innovation in state estimation must be reduced. We use the fault detection function value to calculate the equivalent weight factor. The constructed equivalent weight covariance matrix can be expressed as:

(41) $$\omega_{k}=\left\{\begin{array}{c} 1\;\;\;\;\lambda_{k}\leq T_{\mathrm{down}}\\ T_{\mathrm{down}}/\lambda_{k}\;\lambda_{k}>T_{\mathrm{down}} \end{array}\right.$$

Combined with robust estimation, $\omega_{k}$ is applied to EKF to correct the integrated information. The filter gain, state estimation, and mean square error update equations in the REKF can be expressed as:

(42) $$K_{k}=P_{k/k-1}H_{k}^{T}{\left(H_{k}P_{k/k-1}H_{k}^{T}+R_{k}/\omega_{k}\right)}^{-1}$$

(43) $${\hat{X}}_{k}={\hat{X}}_{k/k-1}+\sqrt{\omega_{k}}K_{k}\left(Z_{k}-h({\hat{X}}_{k/k-1})\right)$$

(44) $$P_{k}=\left(I-\omega_{k}K_{k}H_{k}\right)P_{k/k-1}$$

**Figure 1:**
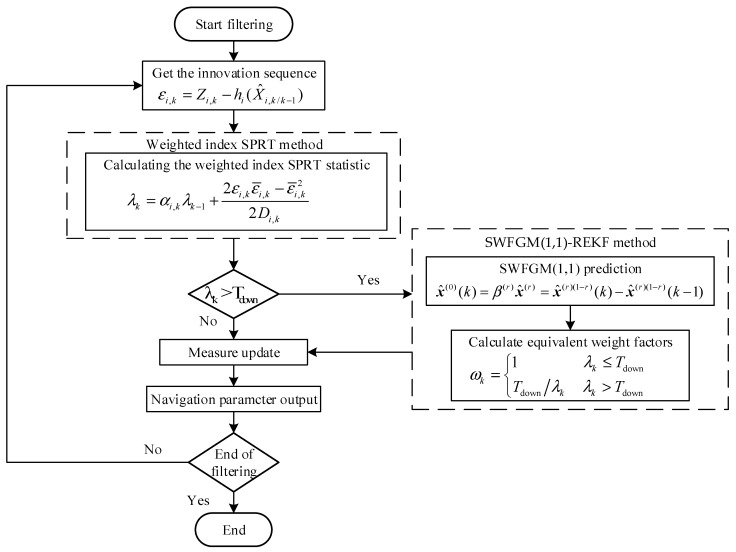
SWFGM(1,1)-REKF flow chart.

In summary, the SWFGM(1,1)-REKF flow is shown in Figure 1, with the main steps:

Step 1: Calculate the innovation, innovation expectation, and innovation covariance.

Step 2: Compute the weighted index SPRT statistic.

Step 3: Judging whether there is a fault in the system according to the threshold value. If there is no fault in the system, update the measurement normally. If there is a fault in the system, the SWFGM(1,1) is used to predict the current time observation. According to the fault detection function, the robust factor is calculated to construct the equivalent weight covariance matrix, and then the measurement is updated.

Step 4: Loop in turn until the end of the algorithm.

## 3. Experimental Results and Analysis

Small amplitude abrupt fault is a typical fault type of MINS/GNSS. Its characteristics have instantaneous occurrence and great change frequency. It is generally caused by hardware damage, signal interference, and other reasons. For example, GNSS receiver clock jump, complex environmental impact, human interference, etc., will cause a small amplitude mutation of measurement information, resulting in a small amplitude mutation failure of the combined system. In order to verify the advancement and reliability of the proposed algorithm, this paper designs simulation and vehicle experiments for experimental verification and analysis.

### 3.1. Simulation Experiments

**Figure 2:**
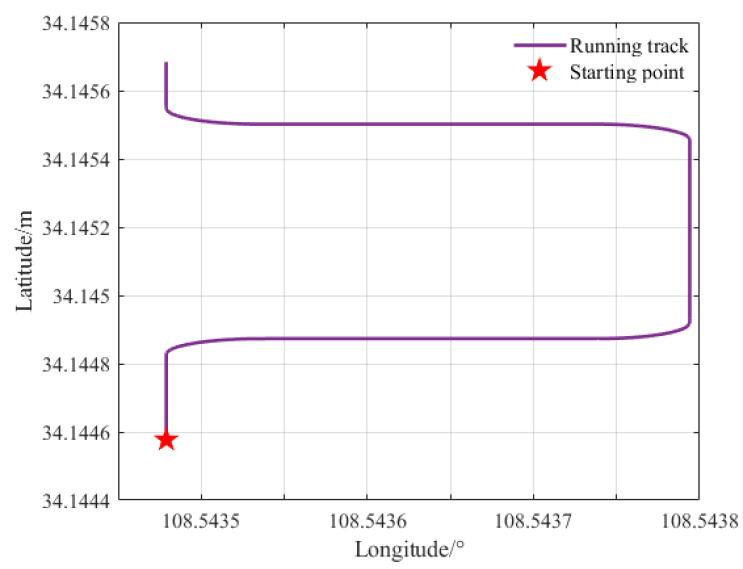
Vehicle trajectory.

In this paper, the PSINS toolbox software (PSINS220204) is employed to simulate the vehicle trajectory. The vehicle operation states include static, uniform velocity, acceleration, deceleration, and so on. The simulated vehicle driving trajectory is illustrated in Figure 2.

The MINS sampling frequency is 200 Hz, and GNSS sampling frequency is 10 Hz. In order to make the experiment more rigorous, we refer to the experimental parameter settings [31]. The system simulation parameters are shown in Table 1. The experimental parameter settings are shown in Table 2.

Three kinds of small amplitude faults are set in the experiment. The three faults were Simulation-a, Simulation-b, and Simulation-c. We used a traditional robust EKF algorithm based on a chi-square test and the robust EKF algorithm based on the sliding window fractional-order grey prediction model proposed in this paper for processing. The chi-square test adopts the sequential form. In order to facilitate writing, the two algorithms are represented by SCSTREKF and SWFGM(1,1)-REKF, respectively. The results of EKF algorithm processing when the data is fault-free are used as the comparison results. Finally, the results of the three algorithms are compared. The settings of the small amplitude faults were as follows.

Simulation-(a): The eastward velocity contained a fault during the period of 301 s–330 s, and the fault was 1 m/s.

Simulation-(b): The northward velocity contained a fault during the period of 561 s–590 s, and the fault was 1 m/s.

Simulation-(c): The altitude contained a fault during the period of 471 s–500 s, and the fault was 50 m.

**Figure 3:**
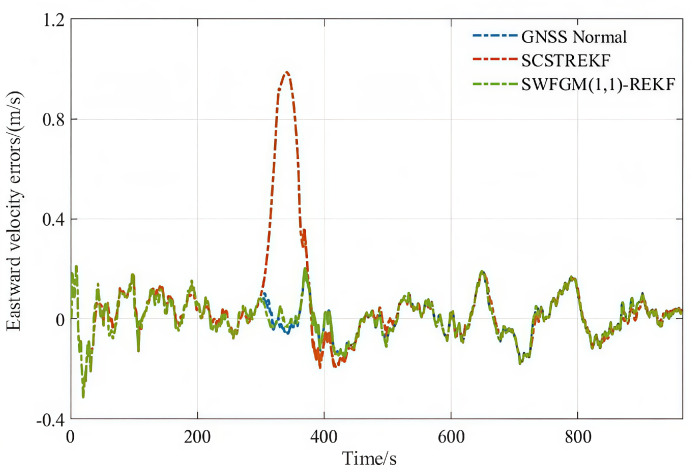
Simulation eastward velocity errors.

**Figure 4:**
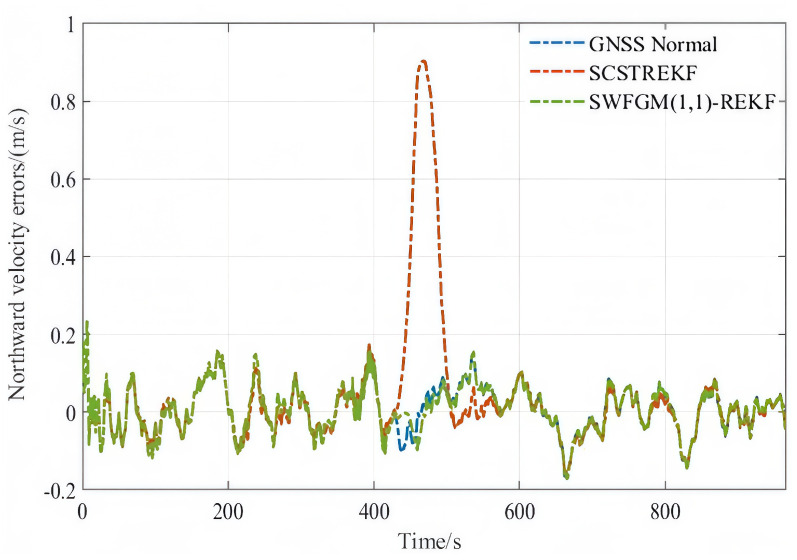
Simulation northward velocity errors.

**Figure 5:**
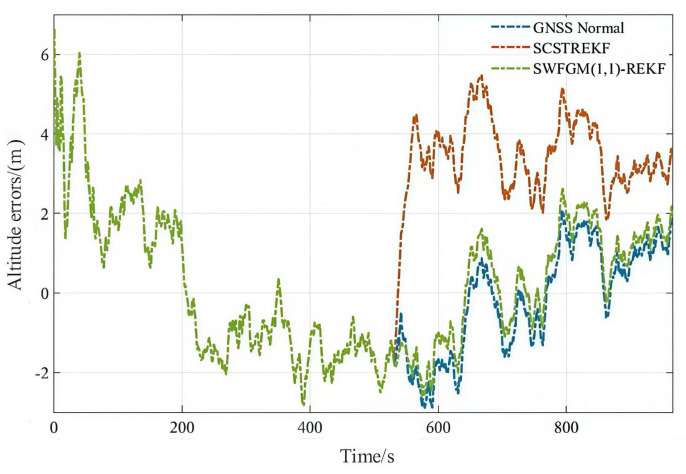
Simulation altitude errors.

The velocity errors and position errors are shown in Figure 3, Figure 4 and Figure 5.

As shown in Figure 3, Figure 4 and Figure 5, when the GNSS signal is disturbed by small amplitude faults, the velocity error curves and the position error curves based on the SCSTREKF algorithm are obviously divergent, and the error values are relatively large. These show that SCSTREKF cannot effectively suppress the impact of faults, which leads to a decrease in MINS/GNSS accuracy. The velocity error curves and position error curves based on SWFGM(1,1)-REKF are relatively convergent, and the error values are small, which are close to the error values when GNSS has no fault. These show that SWFGM(1,1)-REKF can effectively suppress the impact of faults, which improves MINS/GNSS accuracy.

The root mean square error (RMSE) of velocity and position can be summarized as Table 3. $\delta V_{E}$, $\delta V_{N}$, δh represent eastward velocity error, northward velocity error, altitude errors, respectively.

As shown in Table 3, when a GNSS signal is disturbed by small amplitude faults, compared with the traditional SCSTREKF, the accuracy of parameter estimation based on SWFGM(1,1)-REKF is higher. The RMSE of velocity is reduced by one order of magnitude, and the RMSE of position is reduced by 57.7%. Navigation accuracy has been greatly improved.

### 3.2. Vehicle Experiments

**Figure 6:**
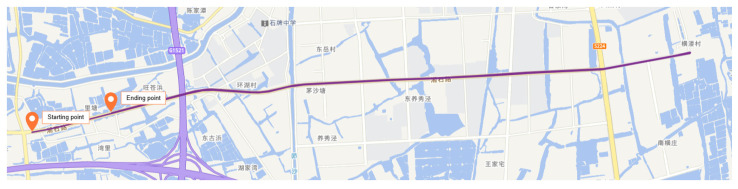
Measured data vehicle trajectory.

In this paper, we use the open MINS/GNSS vehicle integrated navigation experimental data to verify and analyze the algorithm. The running time is 1750 s, the sampling frequency of STIM300 is 200 Hz, and the sampling frequency of GNSS is 10 Hz. The measured data vehicle trajectory is shown in Figure 6.

Three kinds of small amplitude faults are set in the experiment. The three faults were Experiment-a, Experiment-b, and Experiment-c. We use GNSS Normal, SCSTREKF, SWFGM(1,1)-REKF three algorithms for processing. The settings of the small amplitude faults were as follows. Finally, the results of the three algorithms are compared. The settings of the small amplitude faults were as follows.

Experiment-(a) The eastward velocity contained a fault during the period of 711 s–750 s, and the fault was 1 m/s.

Experiment-(b) The northward velocity contained a fault during the period of 1001 s–1030 s, and the fault was 1 m/s.

Experiment-(c) The altitude contained a fault during the period of 801 s–830 s, and the fault was 50 m.

**Figure 7:**
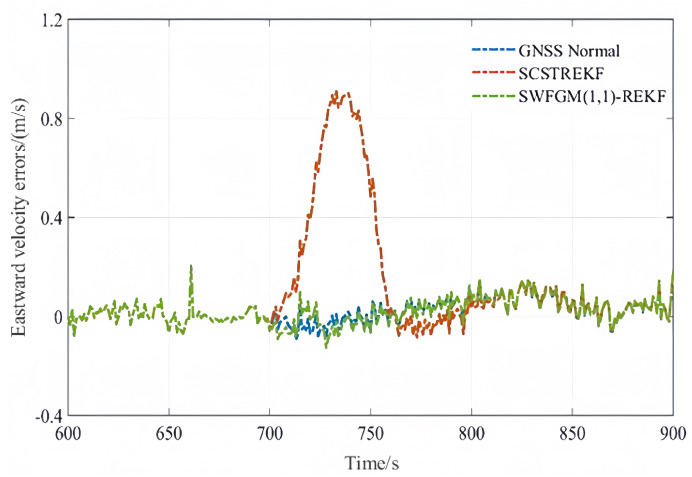
Eastward velocity error.

**Figure 8:**
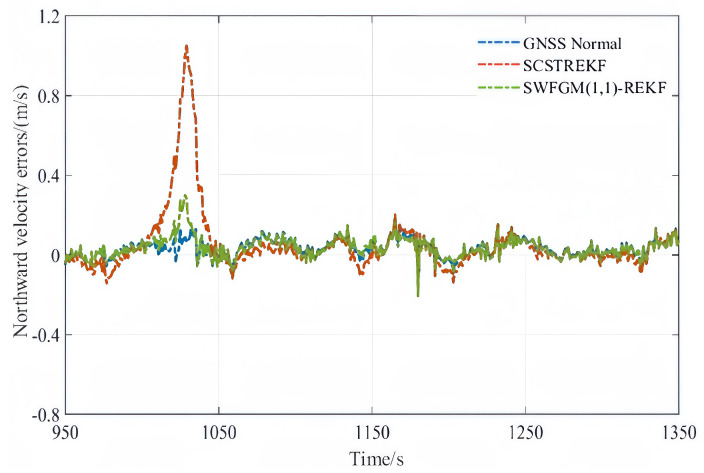
Northward velocity errors.

**Figure 9:**
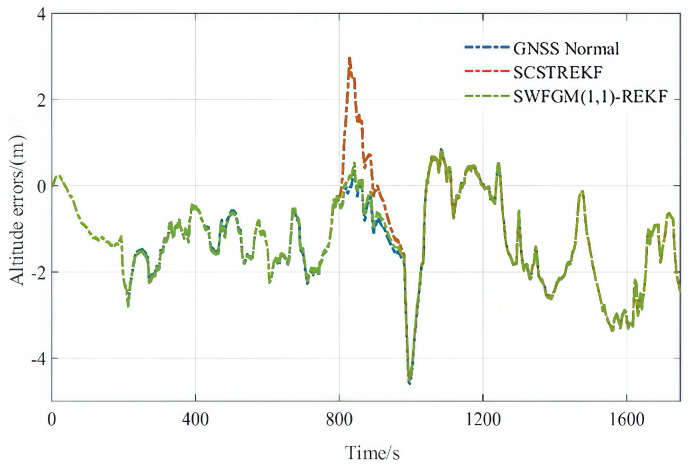
Altitude errors.

The velocity errors and position errors are shown in Figure 7, Figure 8 and Figure 9.

The RMSE of velocity and position can be summarized as Table 4.

As shown in Figure 7, Figure 8 and Figure 9 and Table 4, when GNSS signal is disturbed by small amplitude faults, the velocity error curves and the position error curves based on the SCSTREKF algorithm are obviously divergent, and the error values are relatively large. The velocity error curves and position error curves based on SWFGM(1,1)-REKF are relatively convergent, and the error values are small, which are close to the error values when GNSS has no fault. Compared with the traditional SCSTREKF, the accuracy of parameter estimation based on SWFGM(1,1)-REKF is higher. The RMSE of eastward velocity is reduced by 50.4%, the RMSE of northward velocity is reduced by 70.2%, and the RMSE of position is reduced by 81.0%. Navigation accuracy has been greatly improved.

From the simulation experiments and vehicle experiments, we can see that the proposed SWFGM(1,1)-REKF is superior to the traditional SCSTREKF. In traditional SCSTREKF, the chi-square test statistic is small, which leads to the large weight of fault observations in the measurement update equation, the weak robustness and the low navigation accuracy. SWFGM(1,1)-REKF uses the SWFGM(1,1) model to predict reliable GNSS observations, which reduces the weight of fault observations in the measurement update equation and effectively improves the accuracy of MINS/GNSS.

## 4. Conclusions

To address the issue of reduced accuracy or even divergence in MINS/GNSS integrated navigation systems caused by small amplitude fault in GNSS measurement information, this paper proposes SWFGM(1,1)-REKF. Simulation and vehicle experiment results show that the proposed SWFGM(1,1)-REKF is able to predict reliable GNSS observations to replace fault data, reduce the weight of fault observations in the update equations, and suppress the influence of a fault integrated navigation system. Compared with the traditional robust adaptive algorithm based on the chi-square test, the proposed algorithm improves the velocity and position accuracy. This paper provides a technical path to reconstruct reliable observation information under a high noise background, which provides reference for integrated navigation fault handling. At the same time, it also provides a new idea for the processing of high-noise GNSS time series such as gross error detection and repair in deformation monitoring, high-frequency GNSS seismic waveform recovery, and position prediction when a GNSS signal is interrupted in vehicle navigation.

## Figures and Tables

**Table 1:** System navigation parameter setting.

Equipment	Equipment Parameter	Parameter Setting
INS	Gyro bias instability	0.3°/h
	Gyro noise	0.15${}^{\circ}/\sqrt{h}$
	Accelerometer bias instability	0.05 mg
	Accelerometer noise	0.06 $m/s/\sqrt{h}$
GNSS	Position error	10 m
	Velocity error	0.1 m/s

In Table 1, g represents the gravitational acceleration.

**Table 2:** Experimental parameter settings.

Parameter	Parameter Setting
The missed alarm rate	0.035
The false alarm rate	$8\times 10^{-6}$
The threshold of the detection function	11.7
The size of the sliding window	7
Initial learning rate	0.008
Max epochs	300
Learning factor	2
Inertia weights	0.8–1
The population size of PSO	10

**Table 3:** The RMSE of velocity and position.

Algorithm	$\delta V_{E}$ (m/s)	$\delta V_{N}$ (m/s)	δh (m)
GNSS Normal	0.0449	0.0279	1.2457
SCSTREKF	0.5379	0.4801	2.9456
SWFGM(1,1)-REKF	0.0520	0.0852	1.3557

**Table 4:** The RMSE of velocity and position.

Algorithm	$\delta V_{E}$ (m/s)	$\delta V_{N}$ (m/s)	δh (m)
GNSS Normal	0.0434	0.0565	0.2849
SCSTREKF	0.6043	0.4793	1.5619
SWFGM(1,1)-REKF	0.3000	0.1426	0.2963

## Data Availability

The raw data supporting the conclusions of this article will be made. available by the authors on request. Data download website: www.psins.org.cn/kydm (accessed on 5 January 2023), https://pan.baidu.com/s/1LODpgsLeSLXR8DyehTrxoA (accessed on 5 May 2023).

## References

[1] El-Diasty M. (2020). Evaluation of KSACORS-based network GNSS-INS integrated system for Saudi coastal hydrographic surveys. Geomat. Nat. Hazards Risk.

[2] Mostafa M.M., Moussa A.M., El-Sheimy N., Sesay A.B. (2018). A smart hybrid vision aided inertial navigation system approach for UAVs in a GNSS denied environment. Navig.-J. Inst. Navig..

[3] Alizadeh M., Khoshnood A.M. (2024). Model-aided and vision-based navigation for an aerial robot in real-time application. Intell. Serv. Robot..

[4] Gao G., Gao B., Gao S., Hu G., Zhong Y. (2023). A Hypothesis Test-Constrained Robust Kalman Filter for INS/GNSS Integration with Abnormal Measurement. IEEE Trans. Veh. Technol..

[5] Wang M., Wu W., Zhou P., He X. (2018). State transformation extended Kalman filter for GPS/SINS tightly coupled integration. GPS Solut..

[6] Bando M., Ono Y., Hieida Y., Yamamoto K. (2017). GNSS fault detection with unmodeled error. Adv. Robot..

[7] Yang B., Liu F., Xue L., Shan B. (2023). Fault-Tolerant SINS/Doppler Radar/Odometer Integrated Navigation Method Based on Two-Stage Fault Detection Structure. Entropy.

[8] Wang S., Zhan X., Zhai Y., Liu B. (2020). Fault Detection and Exclusion for Tightly Coupled GNSS/INS System Considering Fault in State Prediction. Sensors.

[9] Gao B., Gao S., Zhong Y., Hu G., Gu C. (2017). Interacting multiple model estimation-based adaptive robust unscented Kalman filter. Int. J. Control Autom. Syst..

[10] Zhang Z., Li Y., Liu Z., Wang S., Xing H., Zhu W. (2023). Enhancing the reliability of shipborne INS/GNSS integrated navigation system during abnormal sampling periods using Bi-LSTM and robust CKF. Ocean Eng..

[11] Gao G., Gao S., Peng X., Hu G. (2020). Fading SPRT method for soft fault diagnosis in SINS/CNS/SRS integrated navigation system. J. Chin. Inert. Technol..

[12] Zhao G., Wang J., Gao S., Jiang Z. (2024). A GNSS/SINS fault detection and robust adaptive algorithm based on sliding average smooth bounded layer width. Meas. Sci. Technol..

[13] Miao Y., Zhou W., Tian L., Cui Z. (2016). Extended robust Kalman filter based on innovation chi-square test algorithm and its application. Geomat. Inf. Sci. Wuhan Univ..

[14] Zhao B., Zeng Q., Liu J., Gao C., Zhu X. (2023). Fault detection and robust adaptive filter algorithm based on smooth bounded layer. J. Chin. Inert. Technol..

[15] Gao G., Gao S., Hong G., Peng X., Yu T. (2020). A Robust INS/SRS/CNS Integrated Navigation System with the Chi-Square Test-Based Robust Kalman Filter. Sensors.

[16] Kassam S.A., Poor H.V. (1985). Robust techniques for signal processing: A survey. Proc. IEEE.

[17] Biswal A., Jwo D.J. (2025). Multi-Kernel Bandwidth Based Maximum Correntropy Extended Kalman Filter for GPS Navigation. Comput. Model. Eng. Sci..

[18] Huang R., Patwardhan S.C., Biegler L. (2013). Robust stability of nonlinear model predictive control with extended Kalman filter and target setting. Int. J. Robust Nonlinear Control.

[19] Tasci L., Tuncez M. (2018). Monitoring of deformations in open-pit mines and prediction of deformations with the grey prediction model. J. Grey Syst..

[20] Kose E., Tasci L. (2019). Geodetic deformation forecasting based on multi-variable grey prediction model and regression model. Grey Syst. Theory Appl..

[21] Jwo D.-J., Biswal A., Mir I.A. (2023). Artificial Neural Networks for Navigation Systems: A Review of Recent Research. Appl. Sci..

[22] Grosu V., David E., Goras L., Pelz G. (2024). On the Modelling Possibilities of Integrated Circuits Behavior Using Active Learning Principles. Rom. J. Inf. Sci. Technol..

[23] Essiz U., Aci C., Sarac E., Aci M. (2024). Deep Learning-Based Prediction Models for the Detection of Vitamin D Deficiency and 25-Hydroxyvitamin D Levels Using Complete Blood Count Tests. Rom. J. Inf. Sci. Technol..

[24] Talebi S., Godsill S., Mandic D. (2023). Filtering Structures for α-Stable Systems. IEEE Control Syst. Lett..

[25] Yan C., Wu L., Liu L., Zhang K. (2020). Fractional Hausdorff grey model and its properties. Chaos Solitons Fractals.

[26] Wang Z. (2024). Research on Vehicle Mounted SINS/GNSS Integrated Navigation Method in Complex Interference Environment. Doctoral Dissertation.

[27] Wang C., Feng W., Huang D. (2025). Adaptive Method for Outlier Detection of GNSS/INS Positioning in Complex Environments. Geomat. Inf. Sci. Wuhan Univ..

[28] Jiang Y., Pan S., Ye F., Gao W., Ma C., Wang H. (2022). Approach for detection of slowly growing fault based on robust estimation and improved AIME. Syst. Eng. Electron..

[29] Meng W. (2015). Grey Prediction Modeling Based on Fractional Order Operators. Doctoral Dissertation.

[30] Cao Y., Bai H., Jin K., Zou G. (2023). An GNSS/INS Integrated Navigation Algorithm Based on PSO-LSTM in Satellite Rejection. Electronics.

[31] Zhong L., Liu J., Yu L., Zhang Z. (2022). Slowly Varying Spoofing Interference Detection Algorithm Based on Adaptive SPRT. J. Signal Process..

